# Student Violence Against Paraprofessionals in Schools: A Social-Ecological Analysis of Safety and Well-Being

**DOI:** 10.3390/bs14121181

**Published:** 2024-12-11

**Authors:** Linda A. Reddy, Andrew H. Perry, Andrew Martinez, Susan D. McMahon, Kailyn Bare, Taylor Swenski, Christopher M. Dudek, Eric M. Anderman, Ron Avi Astor, Dorothy L. Espelage, Frank C. Worrell

**Affiliations:** 1 Graduate School of Applied and Professional Psychology, Rutgers University, New Brunswick, NJ 08901, USA; cdudek@gsapp.rutgers.edu; 2 Department of Educational Studies, The Ohio State University, Columbus, OH 43210, USA; perry.2221@osu.edu (A.H.P.); anderman.1@osu.edu (E.M.A.); 3 New York Center for Justice Innovation, New York, NY 10018, USA; anmartinez@nycourts.gov; 4 Department of Psychology, DePaul University, Chicago, IL 60614, USA; smcmahon@depaul.edu (S.D.M.); kbare1@depaul.edu (K.B.); tswenski@depaul.edu (T.S.); 5 Social Welfare and Education, University of California-Los Angeles, Los Angeles, CA 90095, USA; astor@luskin.ucla.edu; 6 School of Education, University of North Carolina at Chapel Hill, Chapel Hill, NC 27599, USA; espelage@unc.edu; 7 Berkeley School of Education, University of California-Berkeley, Berkeley, CA 94720, USA; frankc@berkeley.edu

**Keywords:** paraprofessionals, special education, violence, social-ecological framework, schools

## Abstract

Violence against teachers has received increasing attention worldwide, with high rates of verbal, threatening, physical, and property violence in schools. Teacher-directed violence contributes to poor mental and physical health, high rates of turnover, and diminished student achievement. Despite these findings, there is a dearth of research on violence experienced by paraprofessionals who play key roles in supporting students with the greatest learning and behavioral needs in schools. Using a sample of 1993 paraprofessionals, this study is one of the first to investigate paraprofessionals’ experiences of violence in school settings. We found that the rate of student violence against paraprofessionals was 37% for property offenses, 49.5% for physical violence, and 54% for verbal and threatening violence. Further, we employed a socio-ecological model of individual, classroom, school, and community factors to predict paraprofessional experiences of violence from students in schools. Negative binomial regression results revealed that student-staff relationship problems and student behavioral concerns were positively related to verbal and threatening, physical, and property violence against paraprofessionals. Paraprofessionals working in elementary schools were more likely to report physical violence compared to those working in middle or high school settings. Implications for research and educational practice are also presented.

## 1. Introduction

Research on school personnel and educator victimization has grown worldwide, reflecting a public health crisis in educational settings [1,2]. The National Center for Educational Statistics [3] School Survey on Crime and Safety indicated that the rates of student verbal and threatening behavior toward classroom teachers almost doubled from 4.8% (2009–2010) to 9.8% (2019–2020). Recent U.S. studies have highlighted that pre-kindergarten (pre-K) through 12th-grade teachers and school staff experience a range of victimization including, but not limited to, verbal aggression, threats, objects thrown, harassment, and physical assaults from students, parents, school leaders, and colleagues [4,5]. Rates of victimization coupled with teacher turnover and shortages have drawn significant attention from educational organizations (e.g., the American Federation of Teachers–AFT) and the National Educational Association–NEA), parents, policymakers, and scholars worldwide [6,7].

The educational role of teachers and school staff is an important predictor of violence in schools [5,8,9]. For example, in a recent national study of 4136 pre-K-12 teachers during the COVID-19 pandemic, special educators were over three times more likely to experience physical violence than general education teachers [5]. Special education teachers and support staff (commonly referred to as paraprofessionals, teacher aides, and teaching assistants) work with students with the most challenging learning and behavioral needs in classroom settings and, as a result, may be at high risk for workplace violence in schools. In particular, paraprofessionals work in close proximity to students who often require behavioral support to maintain their on-task behavior, self-regulation, and academic engagement during learning and social activities (e.g., recess/lunch, extracurriculars). Paraprofessionals are typically low-wage, part-time, or full-time school personnel with high school or college degrees. Whereas classroom teachers are responsible for leading instructional planning and decision-making, paraprofessionals provide instructional and behavioral support under the guidance of teachers, either individually and/or in small groups, which can maximize teacher effectiveness, student behavior, and learning [10,11].

Paraprofessionals are often responsible for implementing behavior interventions and support across settings, such as general education, special education/resource, lunch/recess, and within classrooms and between classroom transitions [5,11,12]. In the U.S, estimates indicate that approximately 40% of states have hired more full-time paraprofessionals than full-time special educators [13], underscoring the value and prevalence of paraprofessionals. Despite paraprofessionals’ contributions to classroom functioning, they receive very limited or no training on effective behavior management and support, which impedes their ability to effectively implement support for students [5,11,12]. Taken together, multiple factors, such as close work proximity to students, behavior support roles, and limited training for behavior management strategies, may substantially increase paraprofessionals’ risks of negative student-staff interactions, student violence, and reduced well-being in schools.

Research on the victimization experiences of classroom paraprofessionals (i.e., teacher aides and teaching assistants) has been very limited, and most studies have been conducted outside the U.S [14,15]. In a statewide study of workplace violence among 6450 Pennsylvania education staff (e.g., teachers, nurses, counselors, and teacher aides) [9], results indicated that 7.8% of school personnel were physically assaulted, and 28.9% experienced verbal and threatening violence during 2009–2010. The results further revealed that special education teachers and teacher aides experienced significantly higher rates of physical and verbal violence than general education teachers; overall, violence was perpetrated by students (95%), males (73%), and in classrooms (62%).

Other investigations of paraprofessionals’ (i.e., teaching assistants) experiences with school violence have been conducted in the United Kingdom (UK). For example, Unison’s [15] national survey of 14,500 school support staff across England, Wales, and Northern Ireland found that approximately 53% of paraprofessionals reported being physically assaulted by students in the previous school year, 53% experienced verbal threats, and 60% reported other forms of verbal abuse. Findings from this large-scale survey suggest that, overall, paraprofessionals experience more student-generated violence than any other school staff member (e.g., teachers and school administrators).

In a recent qualitative study of 16 paraprofessionals, Holt and Birchall [14] explored the nature of physical and psychological abuse from students experienced by paraprofessionals and its relation to gender-based violence in the UK, with nearly all (93%) of paraprofessionals identified as women. Using an incident-based interview format, all interviewed paraprofessionals reported polyvictimization, which included physical, emotional, and verbal abuse. Participants also reported impairment in job-related tasks (e.g., redirecting student attention and behavior, problem-solving peer conflict) and professional confidence, as well as deleterious impacts on mental health (e.g., stress, anxiety, and post-traumatic stress disorder) as a result of workplace violence. The authors concluded that violence against paraprofessionals is understudied due, in part, to feminization and the undervalued nature of its role in UK schools.

In a related line of research, Ravalier and colleagues [16] examined how school climate and organizational conditions, such as job demands, control, and support, can impact paraprofessionals’ well-being. Using a survey, 2957 paraprofessionals from the UK were assessed on the extent to which job demands, control, role clarity, peer and management support, organizational change, relationship quality, and student and parental negative behavior contribute to their work-related stress. The results showed that job demands, control, and disrespectful and threatening behaviors from students and/or parents contributed to increased stress.

The findings from this study and the three investigations on paraprofessional victimization collectively underscore the need to better understand the nature and extent of student violence against paraprofessionals and the possible antecedent factors that predict their well-being and safety in schools. One factor that has been shown to be a powerful predictor in the larger school violence literature is the school climate [17,18]. Although there are many definitions of what constitutes a positive school climate, a few common elements have been positive interpersonal relations among adults (e.g., school leaders, teachers, parents) and students, school safety, and school connections [18]. Indeed, a positive school climate, including greater safety and positive relationships/connections between school stakeholders and students, is associated with less violence directed toward school administrators [19], teachers [5,20], and students [21]. However, research is very limited on how aspects of school climate predict violence against support staff, such as paraprofessionals. To address this gap in the literature, we employed a socio-ecological lens [22,23]. More specifically, we examined individual, school, and community factors as predictors of paraprofessionals’ experiences to guide theory and research on educator-directed violence [5,17,24].

### Purpose of Study

This study is the first national study to date of pre-K through 12th grade paraprofessionals surveyed on their experiences of violence from students in U.S. schools. We examined the contributions of a comprehensive set of socio-ecological variables (individual-, school-, and community-level) using psychometrically-sound scales that assess important factors associated with paraprofessional safety and well-being. Using a socio-ecological measurement framework, the following research questions were addressed: (a) What is the rate of paraprofessional-reported violence from students, including verbal, physical, and property violence?; and (b) To what extent do individual characteristics of paraprofessionals (i.e., race, gender), school (i.e., student-staff relations, student behavioral concerns), and community factors (i.e., urbanicity) predict student violence against paraprofessionals? Based on research on teacher-directed violence, we hypothesized that these factors at the individual, classroom, school, and community levels would predict paraprofessional experiences of student violence in schools [4,5,22].

## 2. Method

### 2.1. Participants

Respondents were 1993 paraprofessionals working in pre-K-12th grade schools in the U.S, recruited from a larger project on violence against pre-K-12 school personnel. The majority of the sample identified as female (*n* = 1797; 90.2%), followed by male (*n* = 180; 9.0%), with 16 respondents (0.8%) declining to provide information on gender. Participants primarily identified as Caucasian/White (*n* = 1315; 66.0%), followed by African-American/Black (*n* = 255; 12.8%), Latinx/Hispanic (*n* = 218; 10.9%), Multiracial (*n* = 101; 5.1%), and other races (*n* = 80; 4%), with 24 respondents (1.2%) declining to provide information on race/ethnicity. Most participants worked in suburban schools (*n* = 812; 40.7%), followed by rural schools (*n* = 628; 31.5%) and urban schools (*n* = 472; 23.7%), with 81 participants (4.1%) declining to provide the urbanicity of their schools. Respondents primarily worked in elementary schools serving pre-K to 6th grades (*n* = 969; 48.6%), followed by high schools (*n* = 369; 18.5%), middle schools (*n* = 315; 15.8%), elementary/middle schools serving pre-K to 9th grades (*n* = 122; 6.1%), and schools serving all grades (pre-K through 12th grades; *n* = 75; 3.8%), with 143 participants (7.2%) declining to provide their school level. Respondents had, on average, 11.80 years of experience in their role (*SD* = 8.87), and they reported that, on average, 71% of their students received free/reduced-price lunch (FRL; *M* = 7.10, *SD* = 2.84; 0–10 scale = 0%–100%).

### 2.2. Measures

A web-based survey was designed and disseminated in collaboration with the APA Task Force on Violence Against Educators and School Personnel representing scholars from universities and organizations, as well as several national organizations, including NEA, AFT, the National Association of School Psychologists (NASP), the National Association of School Social Workers (NASW), and the School Social Work Association of America (SSWAA). Participants completed demographic information and specific study measures via the online survey, taking about 25–30 minutes to complete. As participants were surveyed during COVID-19 (i.e., the 2020–2021 school year), they were asked to complete survey items and scales based on their experiences prior to COVID-19, as well as their experiences after the onset of COVID-19. However, for this study, only pre-COVID-19 responses were analyzed due to school lockdown restrictions in many districts in the country and the temporary suspension of paraprofessional roles in schools during COVID-19. Each survey scale yielded a composite mean score, and the scores were examined for internal consistency (Cronbach’s α and McDonald’s ω) and structural validity (confirmatory factor analysis CFA using maximum likelihood estimation).

#### 2.2.1. School Climate Measures

Participants completed two scales that assessed aspects of school climate. The first, which was the *Student-Staff Relationship Problems Scale* [25,26], assessed negative interactions between school personnel and students (e.g., staff humiliated or shamed students, physically harmed students) on a 5-point frequency scale (0 = *Never*, 1 = *Rarely*, 2 = *Sometimes*, 3 = *Often*, 4 = *Almost always*). The scale consisted of four items, and the total score had adequate internal consistency (α = 0.80; ω = 0.86) and structural validity (e.g., comparative fit index CFI = 0.99, root-mean-square error of approximation RMSEA = 0.09, standardized root-mean-squared residual SRMR = 0.02; [27]. The second, which was the *Student Behavioral Problems Scale* [25,26], assessed student behavior problems in school (e.g., harassment or bullying among students, physical fighting between students, verbal aggression between students, disruptive student behavior) with 4 items on a 4-point severity scale (0 = *Not at all a problem*, 1 = *Mild problem*, 2 = *Moderate problem*, 3 = *Severe problem*). Scale scores had adequate internal consistency (α = 0.88; ω = 0.89) and an acceptable fit to the data (e.g., CFI = 0.94, RMSEA = 0.15, SRMR = 0.05). Construct validity for these measures has been supported in prior studies [4,5], and criterion validity in this study was evidenced by significant correlations in the expected direction with the other constructs.

#### 2.2.2. Victimization Scale

Paraprofessionals completed the *Educator Victimization Scale* [4], which consisted of three subscales that assessed verbal and threatening, physical, and property violence against school staff. Paraprofessionals reported how frequently they experienced each type of violence from each of the four different aggressors (i.e., students, parents/guardians, colleagues, and administrators) during the pandemic on a 6-point scale from *never* to *daily*. In the current study, we focused on violence perpetrated by students. To maximize variability, the responses to each item were dichotomized such that 0 = *never* and 1 = *at least once*. Items were then summed to create a composite score of the number of victimization behaviors that participants experienced.

Verbal and threatening violence were assessed with eight items: obscene remarks or gestures, intimidation, slurs or verbal attacks based on demography, verbal threats, bullying, public humiliation, cyber/internet bullying, and sexual harassment (α = 0.74; ω = 0.78). Physical violence was assessed using three items: objects thrown, ordinary objects used as weapons, and physical attacks (α = 0.81; ω = 0.82). Property violence was assessed using two items: personal or classroom property damaged and personal or classroom property stolen (*r* = 0.45). The subscale scores exhibited good internal consistency and adequate fit to the data as a three-factor measure of violence against paraprofessionals (e.g., CFI = 0.90, RMSEA = 0.10, SRMR = 0.06).

### 2.3. Procedure

Following Institutional Review Board (IRB) procedures approved by University of North Carolina at Chapel Hill, online survey data were collected, coded, and analyzed by the American Psychological Association (APA) Task Force on Violence Against Educators and School Personnel. Participants were contacted via school emails provided by a national marketing firm (MCH Strategic Data) and through national partners who distributed the survey link through email and social media to a subset of their constituents from August 2020 to June 2021. MCH gathers school personnel contact information by conducting website scans of public education data sources and importing this information into a comprehensive database of 5.4 million school staff nationwide. This information was continuously updated and verified to ensure current contact and school information. MCH periodically contacts individuals within this database to allow them to opt out of the list. Participants were emailed with an embedded survey link with the framing of “School Climate and School Safety Survey”, and all persons who received the communication were encouraged to share their experiences, concerns, and recommendations. In the email, the link to the online survey describes the study’s purpose and IRB-approved informed consent procedures. Participant data used in this study were de-identified. No incentives were provided to the participants.

#### Data Analytic Plan

Using SPSS Version 28, three negative binomial regressions were conducted on each of the three dependent variables (verbal/threatening, physical, and property violence). Negative binomial regression was selected as the analytic approach due to the dependent variables being overdispersed. In each model, although variables were entered simultaneously, variables were interpreted in a socio-ecological manner, with paraprofessional characteristics (i.e., gender, race, and years of experience), school climate (i.e., student-staff relationship problems and student behavioral problems), school organizational variables (i.e., percentage of students receiving free/reduced-price lunch FRL, school level), and community characteristics (i.e., urbanicity) being examined consecutively. All reported estimates are standardized coefficients. McFadden’s R^2^ values [28] were calculated to determine the proportion of variance explained in the outcomes of each analysis.

## 3. Results

### 3.1. Preliminary Analyses

Descriptive statistics and correlations can be found in Table 1. The rate of student violence (reporting at least one incident from August 2019 to March 2022) against paraprofessionals was 23.9% for property damage (*M* = 0.49, *SD* = 0.73), 32.5% for physical assault (*M* = 1.01, *SD* = 1.18), and 36.7% for verbal/threatening (*M* = 1.30, *SD* = 1.64). Respondents indicated that student-staff relationship problems (*M* = 1.57, *SD* = 0.58) and student behavioral concerns (*M* = 2.01, *SD* = 0.69) were present, although slightly lower than average on the response scale. Noteworthy associations include positive and meaningful correlations between student and staff relationship problems, student behavioral concerns, and verbal and threatening (*r* = 0.31 and 0.52), physical (*r* = 0.23 and 0.32), and property (*r* = 0.23 and 0.35) violence against paraprofessionals, respectively.

### 3.2. Primary Analyses

To address the research questions in this study, three negative binomial regressions were conducted, examining the role of socio-ecological variables among paraprofessionals on their self-reported victimization (See Table 2 for results). The results are reported separately and organized by dependent variables.

#### 3.2.1. Verbal and Threatening Violence

The results indicated that student-staff relationship problems (Exp(B) = 1.25, *p* < 0.01) and student behavioral concerns (Exp(B) = 2.05, *p* < 0.01) were positively related to verbal and threatening victimization. In other words, the more participants reported relationship problems between staff and students and behavioral concerns among students, the more verbal and threatening victimization behaviors they reported experiencing. No other predictors were found to be significant.

#### 3.2.2. Physical Violence

The results indicated that White paraprofessionals were more likely to experience physical victimization from students compared to Black (Exp(B) = 0.63, *p* < 0.01) and Hispanic (Exp(B) = 0.60, *p* < 0.01) participants. Further, participants from pre-K to 6th-grade elementary schools were more likely to experience physical violence compared to respondents in middle schools (Exp(B) = 0.56, *p* < 0.01) and high schools (Exp(B) = 0.47, *p* < 0.01). Finally, student behavioral concerns were positively related to physical victimization (Exp(B) = 1.66, *p* < 0.01), meaning that the more paraprofessionals reported student behavioral concerns, the more physical violence behaviors they experienced. No other predictors, including student-staff relationship problems, were significant predictors of physical violence.

#### 3.2.3. Property Violence

The results indicated that compared to White paraprofessionals, Latinx/Hispanics (Exp(B) = 0.42, *p* < 0.01) and those reporting “other race” (Exp(B) = 0.38, *p* < 0.05) were less likely to report property violence. Additionally, both student-staff relationship problems (Exp(B) = 1.21, *p* < 0.05) and student behavioral concerns (Exp(B) = 1.71, *p* < 0.01) were positively related to reports of property violence against paraprofessionals, such that increased relationship problems and behavioral concerns were associated with more property violence events.

## 4. Discussion

The current investigation builds on the larger school- and teacher-directed violence literature by presenting results from the first U.S. national survey of paraprofessional experiences with student violence in schools. Paraprofessionals are employed in school systems throughout the world and are viewed as valuable educational support staff for classroom teachers and students with special needs. Paraprofessionals work closely with students with complex learning and behavioral difficulties and are often tasked with implementing behavior interventions and support to meet student needs within and across classroom settings. Their sense of safety and well-being in schools is critically important for forging healthy relationships with students and other school personnel and implementing positive behavior support. However, research on paraprofessional experiences with workplace violence is limited. In this investigation, we examined the prevalence of paraprofessional experiences by type of violence as well as a comprehensive set of socio-ecological factors (i.e., paraprofessional characteristics, classroom, school, and community characteristics) that may predict violence from students in schools. We found that pre-K to 12th grade paraprofessionals reported high rates of violence from their students before the COVID-19 pandemic. For example, over half (54%) of the paraprofessionals surveyed reported being verbally abused by their students. Likewise, half of the paraprofessionals (49.5%) surveyed reported being physically assaulted, and over one-third of the support staff (37%) reported having their property damaged or destroyed. In contrast, in their statewide investigation, Tiesman and colleagues [9] found much lower rates of paraprofessional victimization (24.8% for verbal abuse and 9.8% for physical abuse), reflecting that most paraprofessionals in Pennsylvania did not experience violence from their students. However, the rates in the current study were comparable to or slightly lower than those reported in the UK [15]. Specifically, 60% of paraprofessional participants reported being verbally abused and 53% reported being physically assaulted in the UK. Similar to Tiesman et al., [9], Unison [15] did not survey paraprofessional property damage. Overall, the rates in the current study align with rates reported in the US for classroom teachers prior to the COVID-19 pandemic, which showed that 65% of surveyed teachers reported being verbally attacked or threatened by students, and 42% reported experiencing physical violence from students [4]. These findings have implications for paraprofessional retention and development support.

### 4.1. Socio-Ecological Antecedent Findings

#### 4.1.1. Paraprofessional Personal Characteristics

Using a socio-ecological framework, we also examined the contribution of individual, school, and community characteristics to workplace violence [23]. In summary, we found that personal and contextual factors were associated with an increased risk of violence against paraprofessionals. Specifically, paraprofessional racial/ethnic differences emerged as predictors of violence by student offenders. Paraprofessionals who identified as White were more likely to experience physical abuse than Black and Hispanic paraprofessionals; no differences were found in verbal abuse from students. Similarly, compared to Whites, those identifying as Hispanic and “other race” were less likely to experience property theft or damage from their students.

It is possible that the true rates of Black paraprofessional victimization are not lower, but rather that Black and Hispanic paraprofessionals’ may be under-reporting due to their historical exposure to violence and possible desensitization to victimization in general [29]. Paraprofessionals of color may verbally and nonverbally interact with their students differently than White paraprofessionals or may have higher levels of tolerance for students’ off-task and disruptive behaviors. Although we had not anticipated that White paraprofessionals would report experiencing more violence, it is worth noting that similar trends have been found in the teacher-directed violence literature [30,31], although this body of research has generated conflicting results on race and ethnicity [4]. It is worth noting that as with the teacher workforce, Whites comprised the majority of individuals in this sample of paraprofessionals. Nevertheless, paraprofessional racial/ethnic differences related to workplace violence warrant further investigation to explore possible nuances in paraprofessional beliefs, behavior practices, and student interactions.

Findings of paraprofessional gender (binary) and years of experience in school were not associated with a higher risk of violence (verbal, physical, or property) by students. The current study and other studies–e.g. [14,15]–included large samples of female-identifying paraprofessionals (over 90%), which may possibly mask gender-related differences in reported violence. Likewise, the results suggest that paraprofessionals’ experiences of student violence are consistent across their career stages, which has implications for well-being and professional development.

#### 4.1.2. School Characteristics

In this study, we also found that aspects of the school climate are important conditions associated with paraprofessional-reported violence. These findings are in line with results from previous investigations of violence against educators and school personnel [4,5,19], as well as the larger school violence literature [17,22]. Findings revealed that greater negative staff behavior toward students and student-student behavior problems predicted paraprofessional verbal/threatening and property violence. In turn, student-student behavior problems predicted paraprofessional physical violence. Districtwide use of a multidimensional assessment of school climate may generate valuable data that could guide approaches for promoting healthy student-staff interactions and student prosocial behaviors, as well as mitigating violence against paraprofessionals and other stakeholders in schools.

School factors were found to predict paraprofessional student violence. Specifically, we found that paraprofessionals working in elementary (pre-K-6th grade) schools were more likely to experience physical violence from students than middle and high school paraprofessionals. Similar school-level differences were reported in a larger study of student violence against educators and school personnel [4]; younger students exhibit less refined self-control, regulatory processes, and problem-solving and, as a result, may be more disruptive and aggressive in the learning environment [2,32]. Additionally, we found that being employed in schools to serve higher percentages of students receiving free and reduced lunch (FRL) was not related to paraprofessionals’ experiences of violence, suggesting that lower socioeconomic settings defined by high rates of FRL in schools did not represent a risk factor. One explanation is that FRL is a contextual measure and often one dimension of neighborhood disadvantage. Thus, FRL alone may not directly relate to the factors that trigger student violence. It is also possible that student violence against paraprofessionals may be influenced by more proximal school factors, such as school support and staffing ratios. Nevertheless, this finding contrasts with studies of teacher-directed violence, where schools in South Korea with higher percentages of FRL had a greater risk of verbal and property violence for teachers and a 35% lower risk of misbehavior [33].

#### 4.1.3. Community Characteristics

Finally, in the community context, urbanicity was not found to be a predictor of paraprofessional violence from students. These findings suggest that paraprofessional safety and well-being are consistent across rural, suburban, and urban contexts. Interestingly, urbanicity has been found to be a meaningful predictor of teacher-directed violence, e.g. [20,34]. It is possible that urbanicity is less central to student violence against paraprofessionals, and other more proximal structural supports are more central to their experiences. More research is needed to further investigate the role of school and community factors in protecting paraprofessionals from violence.

### 4.2. Limitations

The current investigation has several limitations. First, it is possible that paraprofessionals who volunteered to complete this survey may have had unique experiences of school and/or community violence. Some participants may have felt more comfortable with the anonymous nature of the web-based survey and were more likely to participate, whereas others may have felt less comfortable completing the survey due to fear of exposure. Second, the study was cross-sectional, which precludes causal inferences of the antecedent predictors of violence type. Third, the participants were largely female and White, which may limit the generalizability to the violent experiences of male paraprofessionals and those who are racially diverse. Fourth, this study was conducted before the COVID-19 pandemic, and the findings cannot be generalized to other periods, such as during or after pandemic restrictions. Fifth, the data used in this study are exclusively self-reported and may not fully capture paraprofessionals’ experiences with students in school settings. Sixth, although ecological factors were examined as possible predictors, other individuals (e.g., intersectionality), schools (e.g., staff turnover and school resource office practices), and community contexts (neighborhood danger indices) should be assessed in future investigations of this group of school personnel. In summary, these limitations do not represent fatal flaws for the current investigation, but opportunities for future research.

### 4.3. Directions for Educational Practice and Research

The findings of this study offer directions for school practice and practice-based research. First, school systems can benefit from adopting comprehensive, efficient assessments of wellness (e.g., stress, burnout), safety concerns (e.g., victimization), and school climate across school stakeholders, including school leaders, teachers, support staff (e.g., paraprofessionals, bus drivers, administrative staff, resource officers), and students [4]. To this end, data sources that offer valuable insights for paraprofessional, school, and districtwide safety issues can help inform action plans for improving safety and climate that are tailored to different stakeholders and settings.

Second, this study highlights the importance of school climate as a predictor of paraprofessionals’ experiences of violence. School personnel- and student-initiated harm to students relates to paraprofessional harm, illustrating a reciprocal and dynamic process. School climate is one of the strongest predictors of school violence among students [18,22], and this study extends these findings to school paraprofessionals, underscoring the importance of school climate for the broader school community. Future school system practices and research can be informed by school improvement frameworks, such as The National School Climate Standards, which delineate specific steps toward improving school climate.

Third, findings from this study, such as school staff humiliating students, underscore that paraprofessionals and the broader school community need tailored professional development to expand their knowledge and improve the implementation skills of behavioral interventions aligned with student needs. School professional development strategies can include preventative practices such as trauma-informed practices [35] and intervention strategies that educators and school personnel identify as training needs (e.g., de-escalation, social-emotional learning, trauma-informed practices, restorative justice practices, and working with diverse groups) [4].

### 4.4. Directions for Educational Research

This is the first national study to examine predictors of violence directed against paraprofessionals, and strong empirical grounding is needed to move this field forward. As such, nationally representative studies on paraprofessional violence are warranted. These can include studies that further establish prevalence rates across different paraprofessional roles and educational contexts. Doing so would further contextualize the findings of the current study and generalize the findings to a larger population of paraprofessionals.

In addition, studies are needed to examine the strategies schools use to address and prevent victimization and how they affect paraprofessionals. Past research has shown that school safety strategies endorsed by teachers do not necessarily align with effective strategies informed by research [22], and understanding paraprofessionals’ views and the use of effective strategies can serve as a starting point. Rigorous experimental designs are also needed to better understand the impact of effective interventions.

A more complete picture of paraprofessional violence can be achieved through mixed-method studies incorporating multiple data sources, school stakeholders, and the assessment of contextual and situational factors accompanying violence. This could include interviews, focus groups, and observations to capture the experiences, perspectives, and recommendations of paraprofessionals.

Finally, studies with repeated measures research designs would help identify correlates of violence and potential malleable risk factors that can serve as targets for school-based interventions. Longitudinal analyses could help ascertain the long-term impact of violence experiences on paraprofessional well-being and retention and the effectiveness of targeted interventions.

## 5. Conclusions

Paraprofessionals are increasingly being hired to serve the most socially and behaviorally vulnerable students in schools and are at risk of workplace violence and diminished well-being. However, research on paraprofessional-directed violence by students is limited. This study serves as a first step to address this gap in school violence literature by examining the nature and extent of paraprofessional victimization among students and possible ecological predictors that may contribute to their safety and well-being in schools. The findings underscore that paraprofessionals are at a high risk of verbal, physical, and property violence from students (rates ranged from 37% to 54%). Also, we found that factors such as paraprofessional race/ethnicity, negative interactions between school personnel and students, student-to-student behavior problems, and school level (elementary grades) are meaningful predictors of paraprofessional violence from students. Specifically, school climate characteristics, such as negative staff-student interactions and student behavioral concerns, were positively related to verbal and threatening, physical, and property violence against paraprofessionals. It is our hope that this study fosters additional research on these support staff and possible avenues for comprehensive training and additional support [11].

## Figures and Tables

**Table 1:** Descriptive Statistics and Correlations for Continuous Variables.

	*M*	*SD*	Range	1	2	3	4	5	6	7
1. Years of experience	11.80	8.87	1–45	-						
2. FRL	7.10	2.84	10%–100%	0.05	-					
3. Student-staff relationship problems	1.57	0.58	1–5	0.07 *	<0.01	-				
4. Student behavioral concerns	2.01	0.69	1–4	0.04	0.16 **	0.40 **	-			
5. Verbal and threatening violence	1.30	1.64	0–8	0.03	0.05	0.31 **	0.52 **	-		
6. Physical violence	1.01	1.18	0–3	0.02	0.02	0.23 **	0.32 **	0.51 **	-	
7. Property violence	0.49	0.73	0–2	0.06	0.02	0.23 **	0.35 **	0.55 **	0.61 **	-

Notes. *N* = 1993. * *p* < 0.05. ** *p* < 0.01. FRL = Percentage of students receiving free/reduced price-lunch (0–10 = 0%–100%).

**Table 2:** Negative Binomial Regression Results of Social-Ecological Predictors of Paraprofessional Violence.

		Violence Against Paraprofessionals								
		Verbal/Threatening			Physical			Property		
Predictors (Reference Categories)		Exp(B)	S.E.	*p*	Exp(B)	S.E.	*p*	Exp(B)	S.E.	*p*
Paraprofessional Characteristics:										
Gender (Female)										
	Male	0.86	0.14	0.29	0.98	0.16	0.91	0.87	0.21	0.48
Race (White)										
	Black	0.86	0.13	0.26	0.63 *	0.16	0.01	0.78	0.19	0.21
	Hispanic	0.82	0.16	0.20	0.60 *	0.18	0.01	0.42 **	0.29	<0.01
	Multiracial	1.22	0.18	0.26	1.04	0.20	0.87	0.92	0.28	0.76
	Other race	0.74	0.24	0.19	0.89	0.26	0.65	0.38 *	0.45	0.03
Years of teaching experience		1.00	<0.01	0.42	0.99	0.01	0.15	1.00	0.01	0.74
School Climate Characteristics:										
Student-staff relationship problems		1.25 **	0.07	<0.01	1.14	0.08	0.09	1.21 *	0.09	0.04
Student behavioral concerns		2.05 **	0.06	<0.01	1.66 **	0.07	<0.01	1.71 **	0.09	<0.01
School Organizational Characteristics:										
FRL		1.00	0.01	0.89	0.99	0.02	0.57	0.99	0.02	0.60
School level (pre-K-6th)										
	pre-K-9th	0.67	0.20	0.05	0.70	0.20	0.07	0.76	0.26	0.30
	Middle school	0.89	0.11	0.28	0.56 **	0.13	<0.01	0.80	0.15	0.15
	High school	1.11	0.10	0.29	0.47 **	0.14	<0.01	0.76	0.15	0.07
	All grades	1.35	0.21	0.15	0.97	0.24	0.68	1.09	0.29	0.77
Community Characteristic:										
Urbanicity (Suburban)										
	Rural	0.87	0.10	0.18	0.92	0.11	0.47	1.01	0.14	0.97
	Urban	0.95	0.10	0.59	1.05	0.11	0.69	1.02	0.14	0.90
McFadden’s R^2^		0.08			0.08			0.08		

Notes. *N* = 1993. FRL = Percent of students receiving free/reduced-price lunch. * *p* < 0.05, ** *p* < 0.01.

## Data Availability

The data are not currently available for public access due to the size of the data sets, which are currently undergoing ongoing data cleaning, organizing, scale refinement, and scale validation. The study materials are available upon request.
